# Relationship between serum bilirubin levels and mortality in patients on peritoneal dialysis

**DOI:** 10.1080/0886022X.2019.1628062

**Published:** 2019-06-26

**Authors:** Xiaojiang Zhan, Mei Yang, Yanbing Chen, Caixia Yan, Yifan Wang, Qing Zhao, Qinkai Chen, Li Zhang

**Affiliations:** Department of Nephrology, The First Affiliated Hospital of Nanchang University, Nanchang, Jiangxi, China

**Keywords:** Peritoneal dialysis, total bilirubin (TBil), indirect bilirubin (IBil), mortality

## Abstract

**Background:** Studies have shown that the serum total bilirubin (TBil) is associated with the mortality of the general population and of hemodialysis patients. However, few studies have examined the associations of the direct bilirubin (DBil) and indirect bilirubin (IBil) with the mortality of peritoneal dialysis (PD) patients.

**Methods:** This was a retrospective cohort study. Clinical and laboratory data were collected from 740 PD patients. The primary endpoint was 5-year all-cause mortality. Survival analysis was performed using the Kaplan–Meier method with the log-rank test. The mortality hazard ratio was evaluated using Cox regression models.

**Results:** Among the 740 PD patients, the mean age was 49.9 ± 15.0 years, 54.9% were men, and 20.3% had diabetes. During the median follow-up period of 28 months (interquartile range, 14–41 months), 178 patients died. Kaplan–Meier analysis revealed that all-cause mortality was higher in the patients in the higher TBil group than in the lower TBil group (25.6% vs. 18.3%, *p* = .017) and in patients in the higher IBil group than in the lower IBil group (24.3% vs. 19%, *p* = .026). Multivariate analysis showed that compared with the lower TBil group, the 5-year mortality risk was higher in the higher TBil group (HR = 1.69, 95% CI: 1.11–2.56, *p* = .014). Similarly, there was a 56% higher risk of 5-year mortality in the higher IBil group than in the lower IBil group (HR = 1.56, 95% CI: 1.04–2.34, *p* = .032). However, no such associations were observed between the DBil and the mortality risk.

**Conclusions:** The baseline serum TBil and IBil levels were significantly associated with 5-year all-cause mortality among PD patients.

## 1. Introduction

Peritoneal dialysis (PD) is one of the main treatments for end-stage renal disease (ESRD) patients. At present, more than 272 000 ESRD patients receive PD worldwide, which accounts for approximately 11% of dialysis patients [1]. Compared with the general population and predialysis chronic kidney disease patients, PD patients have increased levels of oxidative stress (OS), which is associated with increased cardiovascular risk [2]. Moreover, previous studies have shown that cardiovascular disease (CVD) accounts for the most common cause of death among patients requiring dialysis [3,4].

Bilirubin, as the end-product of heme metabolism, has been discovered to have antioxidant properties [5–7], whether conjugated, unconjugated [8], free or protein bound [7]. Unconjugated bilirubin functions intracellularly, as a potent inhibitor of NADPH oxidase complexes and albumin-bound bilirubin contributes significantly to the oxidant scavenging activity of plasma, so bilirubin can act as an oxidant scavenger [9].

Current evidence indicates that mildly elevated bilirubin is associated with protection from cardiovascular disease and from all-cause mortality in adults [10]. Moreover, previous studies found that the elevation of serum total bilirubin (TBil) or indirect bilirubin (IBil) levels are independent protective factors for patients with type 2 diabetes mellitus and patients with chronic kidney disease (CKD) in stages 1–5 [11,12]. Similarly, another study showed that a low-serum TBil concentration is a risk factor for the development of CKD [13]. However, studies have also reported that a high serum bilirubin level is associated with a high mortality rate among jaundice patients [8] and high-serum bilirubin has well-documented neurotoxic effects in infants [10]. Furthermore, among chronic hemodialysis (HD) patients, a large retrospective long-term cohort study with 115 535 HD patients showed that a high TBil level is associated with a significantly higher mortality rate [14]. However, a longitudinal cohort study of the general population showed that neither serum TBil nor IBil levels were significantly associated with the incidence of CKD [15]. Thus, there have been conflicting results regarding the effects of bilirubin on CKD and in dialysis patients. In addition, few studies have focused on the effect of bilirubin in PD patients; only one study reported that a slightly elevated TBil level is a protective factor for PD patients and is a useful biomarker for predicting the clinical course of PD patients [16]. However, this study only focused on TBil without distinguishing between direct bilirubin (DBil) and IBil.

Therefore, this study aimed to evaluate the relationship of the levels of the three types of serum bilirubin (TBil, DBil and IBil) with the 5-year all-cause mortality of PD patients.

## 2. Materials and methods

### 2.1. Study population and data collection

All incident patients who used PD as their first renal replacement treatment modality from the PD Center of the First Affiliated Hospital, Nanchang University, Jiangxi, China between 1 November 2005 and 28 February 2017 were screened. Patients who were aged ≥18-years-old at the start of PD and survived for at least 90 days from their initial PD therapy were enrolled. Patients who were catheterized in other hospitals shifted from permanent HD or failed renal transplantation therapy were excluded. Patients who met the following criteria were also excluded: (1) patients with hepatitis B virus or hepatitis C virus; and (2) patients with serum bilirubin levels higher than the upper limit of the normal range, who did not have a known baseline bilirubin level, or who showed abnormal liver enzyme tests (alanine aminotransferase (ALT) > 40 IU/L or aspartate aminotransferase (AST) > 40 IU/L). The study was conducted in compliance with the ethical principles of the Declaration of Helsinki [17]. Moreover, written informed consent was obtained from each patient involved.

The primary endpoint of this study was 5-year all-cause mortality. All patients were followed up until the cessation of PD, death, or 31 May 2017. Baseline demographic data were collected, including age, sex, the primary cause of ESRD, the presence of diabetes and CVD. Clinical and biochemical data were collected at the initiation of PD, including blood pressure, estimated glomerular filtration rate (eGFR), hemoglobin, serum albumin, blood urea nitrogen, total cholesterol (CHOL), triglyceride (TG), ALT, AST, TBil, DBil, serum creatinine (SCr), blood uric acid, serum phosphorus (P), serum calcium (Ca), intact parathyroid hormone (iPTH), and total urea clearance (tKt/V). The IBil level was calculated using the following formula: IBil  =  TBil − DBil. All baseline data were obtained during the first 1–3 months of PD. Baseline residual renal function was assessed by eGFR using the Chronic Kidney Disease Epidemiology Collaboration creatinine equation [18].

### 2.2. Statistical analysis

We calculated the area under the receiver operating characteristic (ROC) curve and selected the optimal cut-off value with the maximum sum of specificity and sensitivity, and we divided the patients into two groups according to the cut-off values of the three types of serum bilirubin levels: the lower bilirubin group (TBil ≤ 3.3 µmol/L, IBil ≤ 1.5 µmol/L, and DBil ≤ 1.4 µmol/L) and higher bilirubin group (TBil > 3.3 µmol/L, IBil > 1.5 µmol/L, and DBil > 1.4 µmol/L). Numeric parameters are presented as the mean ± standard deviation and categorized data are presented as the number and percentage *n* (%). Differences between groups were assessed using chi-square or Student’s *t* tests or the Kruskal–Wallis test. Survival analysis between the groups was performed using the Kaplan–Meier method with the log-rank test. The mortality hazard ratio was evaluated using Cox proportional hazards models. Sensitivity analyses by excluding patients with follow-up periods less than 1 year were performed to assess the robustness of the results. The censored data included those of patients who switched to HD, who underwent renal transplantation, who transferred to another center, who were lost to follow-up, or who were still at our PD center on 31 May 2017. Covariates with *p* < .1 in the univariate Cox analyses or that were thought to be clinically significant were used for multivariate Cox proportional hazards regression analysis. The results are expressed as the hazard ratios (HR) and 95% confidence interval (95% CI). All descriptive and multivariate analyses were conducted using SPSS version 21.0 (SPSS, Inc., Chicago, IL, USA). A value of *p* < .05 was considered statistically significant.

## 3. Results

### 3.1. Baseline patient characteristics

**Figure 1.**
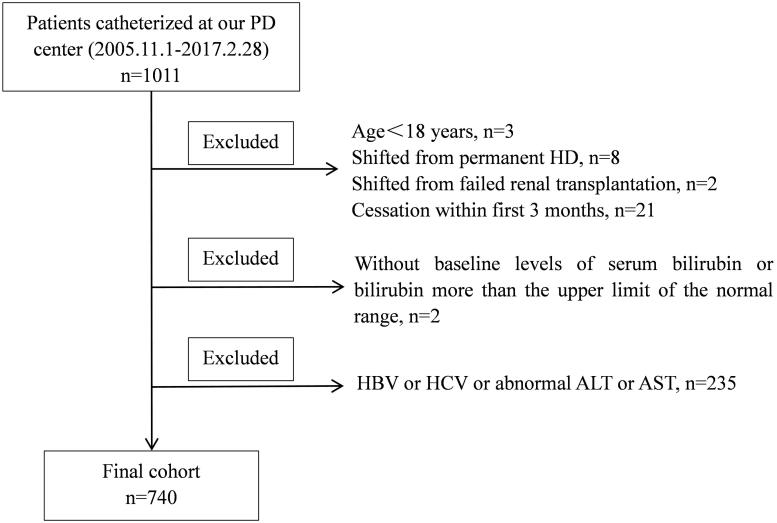
Study flow chart. ALT: alanine aminotransferase; AST: aspartate aminotransferase; HBV: hepatitis B virus; HCV: hepatitis C virus; HD: hemodialysis; PD: peritoneal dialysis.

All PD patients (*n*  =  1011) were recruited and monitored at our hospital. Out of 1011 patients, 34 patients were excluded based on our exclusion criteria, as follows: 3 patients were under 18 years of age, 2 patients were transitioned from a failed renal transplantation, 8 patients were transitioned from permanent HD, and 21 patients received PD for less than 3 months. The remaining 977 patients were enrolled. Of the 977 patients, patients were excluded for the following reasons: 2 patients with serum bilirubin levels greater than the upper limit of the normal range or who did not have baseline serum bilirubin measurements; 83 patients with ALT > 40 IU/L or AST > 40 IU/L; and 152 patients with hepatitis B virus or hepatitis C virus. Finally, 740 PD patients were enrolled in the study (Figure 1). As shown in Table 1, in this study, the mean (± SD) age of the patients was 49.9 ± 15.0 years, 54.9% of the patients were men and 20.3% of the patients were diabetic. The leading cause of ESRD was chronic glomerulonephritis, which accounted for 63.6% of the group, followed by diabetic nephropathy (17.2%) and hypertension (12.4%).

**Table 1.** Baseline characteristics of the PD patients according to the serum bilirubin.

Variables	Whole Group	TBil (µmol/L)		*p* Value	IBil (µmol/L)		*p* Value	DBil (µmol/L)		*p* Value
		≤3.3	>3.3		≤1.5	>1.5		≤1.4	>1.4	
Number	740	349	391		295	445		294	446	
Age (years)	49.9 ± 15.0	48.7 ± 14.3	50.9 ± 15.4	.043	49.9 ± 14.4	49.8 ± 15.3	.954	48.6 ± 15.0	50.7 ± 14.9	.065
Male, *n* (%)	406 (54.9)	175 (50.1)	231 (59.1)	.015	157 (53.2)	249 (56.0)	.464	139 (47.3)	267 (59.9)	.001
DM, *n* (%)	150 (20.3)	77 (22.1)	73 (18.7)	.252	78 (26.4)	72 (16.2)	.001	44 (15.0)	106 (23.8)	.004
HTN, *n* (%)	546 (73.8)	252 (72.2)	294 (75.2)	.357	216 (73.2)	330 (74.2)	.777	208 (70.7)	338 (75.8)	.127
CVD, *n* (%)	78 (10.5)	33 (9.5)	45 (11.5)	.364	29 (9.8)	49 (11.0)	.609	25 (8.5)	53 (11.9)	.143
PD duration (m)	30.6 ± 20.6	31.8 ± 21.9	29.6 ± 19.2	.15	32.8 ± 20.5	29.2 ± 20.5	.019	29.7 ± 23.8	31.2 ± 18.1	.005
Hemoglobin (g/L)	78.1 ± 16.1	73.2 ± 14.6	82.4 ± 16.1	<.001	74.3 ± 14.6	80.6 ± 16.5	<.001	73.0 ± 15.9	81.4 ± 15.3	<.001
Blood platelets (g/L)	170.7 ± 69.5	177.0 ± 69.4	165.2 ± 69.2	.021	181.0 ± 71.2	163.9 ± 67.6	.001	175.2 ± 65.9	167.8 ± 71.7	.153
ALT (U/L)	13.7 ± 8.1	13.9 ± 8.3	13.4 ± 7.9	.464	14.1 ± 8.6	13.4 ± 7.8	.234	13.7 ± 8.2	13.6 ± 8.1	.902
AST (U/L)	17.8 ± 6.2	17.6 ± 6.1	18.0 ± 6.3	.426	17.7 ± 6.1	17.9 ± 6.4	.616	17.7 ± 6.3	17.9 ± 6.2	.746
Albumin (g/L)	35.5 ± 5.2	34.4 ± 5.2	36.4 ± 5.1	<.001	34.1 ± 5.2	36.4 ± 5.1	<.001	35.0 ± 5.2	35.8 ± 5.2	.038
Uric acid (µmol/L)	448.0 ± 130.1	445.8 ± 130.8	450.1 ± 129.6	.656	453.6 ± 132.5	444.4 ± 128.8	.344	447.7 ± 135.3	448.2 ± 126.7	.961
Creatinine (µmol/L)	739.9 ± 302.7	762.4 ± 318.6	719.8 ± 286.7	.056	744.6 ± 314.3	736.8 ± 295.1	.732	787.8 ± 329.9	708.3 ± 279.3	.984
Urea nitrogen (mmol/L)	23.9 ± 9.4	25.4 ± 10.1	22.50 ± 8.62	<.001	25.1 ± 10.1	23.1 ± 8.9	.011	25.6 ± 10.6	22.7 ± 8.4	.011
Total CHOL (mmol/L)	4.2 ± 1.2	4.3 ± 1.3	4.1 ± 1.1	.39	4.3 ± 1.3	4.2 ± 1.2	.348	4.3 ± 1.3	4.1 ± 1.1	.018
TG (mmol/L)	1.5 ± 1.0	1.6 ± 1.1	1.4 ± 0.8	.026	1.6 ± 1.2	1.4 ± 0.8	.104	1.6 ± 0.9	1.4 ± 1.0	.134
Ca (mmol/L)	2.20 ± 0.3	1.9 ± 0.3	2.0 ± 0.2	<.001	1.9 ± 0.3	2.0 ± 0.3	<.001	1.9 ± 0.3	2.00 ± 0.3	.027
P (mmol/L)	1.9 ± 0.5	1.9 ± 0.6	1.8 ± 0.5	<.001	1.9 ± 0.6	1.8 ± 0.5	.044	1.9 ± 0.6	1.8 ± 0.5	.129
iPTH (pg/mL)	252.3 ± 203.1	276.3 ± 207.0	231.3 ± 197.6	.005	261.2 ± 198.9	245.9 ± 206.1	.346	295.2 ± 221.9	226.6 ± 186.6	.152
eGFR (ml/min.1.73 m^2^)	4.0 ± 3.3	3.4 ± 2.6	4.4 ± 3.8	.002	3.8 ± 2.9	4.1 ± 3.6	.607	3.5 ± 3.3	4.2 ± 3.3	.607
tKt/V	2.3 ± 1.6	2.4 ± 2.0	2.3 ± 1.1	.257	2.4 ± 2.1	2.3 ± 1.2	.173	2.4 ± 1.9	2.3 ± 1.4	.369

TBil: total bilirubin; IBil: indirect bilirubin; DBil: direct bilirubin; DM: diabetes mellitus; HTN: hypertension; CVD: cardiovascular disease; ALT: alanine aminotransferase; AST: aspartate aminotransferase; CHOL: cholesterol; TG: triglyceride; Ca: serum calcium; P: serum phosphorus; iPTH: intact parathyroid hormone; eGFR: estimated glomerular filtration rate; tKt/V: total urea clearances; PD: peritoneal dialysis.

### 3.2. Relationship among the baseline serum levels of the three types of bilirubin (TBil, DBil and IBil) and the clinical parameters in PD patients

As shown in Table 1, the patients were divided into the following two groups according to the TBil cut-off value (3.3 µmol/L): the lower TBil group (≤3.3 µmol/L) and higher TBil group ( > 3.3 µmol/L). The age, male ratio, eGFR level and serum levels of hemoglobin, albumin, and Ca were higher, and the serum levels of TG, urea nitrogen, P and iPTH were lower, in the higher TBil group than in the lower TBil group. Likewise, the patients with a higher level of IBil had a lower diabetic ratio, increased levels of hemoglobin, albumin, and Ca, and decreased levels of urea nitrogen and P compared to those in patients with a lower level of IBil. The levels of hemoglobin, albumin, and Ca were positively associated with the levels of the three types of bilirubin, while the level of urea nitrogen was negatively associated with the levels of the three types of bilirubin.

### 3.3. Association between serum bilirubin levels and the 5-year all-cause mortality of PD patients

**Figure 2.**
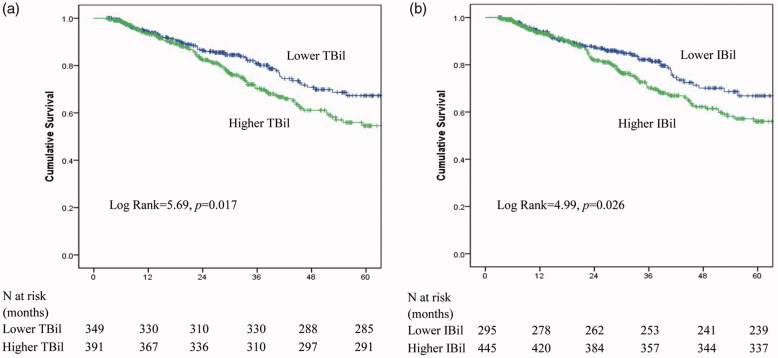
Kaplan-Meier curve of all-cause mortality. (a) Two groups with either lower or higher TBil levels. (b) Two groups with either lower or higher serum IBil levels.

Out of 740 patients, a total of 178 patients died during the median follow-up period of 28 months (interquartile range, 14–41 months). By the end of the study, 41 (5.5%) patients underwent renal transplantation, 123 (16.6%) patients were shifted to HD, 2 (0.3%) patients were transferred to other PD centers, 20 (2.7%) patients were lost to follow-up and the remaining 376 (50.8%) patients were still being followed at our PD center. Of the 178 deaths, 96 (53.9%) were caused by CVD, 16 (9.0%) were caused by infectious disease, 2 (1.1%) were caused by malignancy, 13 (7.3%) were caused by cachexia, 16 (9.0%) were caused by other reasons and 35 (19.7%) were due to unknown causes. The correlation between 5-year all-cause mortality and bilirubin levels was evaluated by using Kaplan–Meier methods and the results are shown in Figure 2. The results revealed that all-cause mortality was higher in patients in the higher TBil group than in patients in the lower TBil group (25.6% vs. 18.3%, *p* = .017) (Figure 2(a)) and in patients in the higher IBil group than in patients in the lower IBil group (24.3% vs. 19%, *p* = .026) (Figure 2(b)). Furthermore, the association among the three types of bilirubin and the 5-year all-cause mortality of the PD patients was evaluated by using Cox regression analysis. As shown in Table 2, compared with the lower TBil group, the HR (and 95% CI) of the 5-year mortality for the higher TBil group was 1.46 (1.07–2.00), *p* = .018. The corresponding HR (95% CI) for IBil was 1.442 (1.044–1.991), *p* = .026. After adjusting for age, sex, diabetes, cardiovascular disease, hypertension, eGFR, albumin level, and hemoglobin, the mortality rate was higher in the higher TBil group than in the lower TBil group (HR = 1.69, 95% CI: 1.11–2.56, *p* = .014). Similarly, there was a 56% higher risk of 5-year mortality in the higher IBil group than in the lower IBil group (HR = 1.56, 95% CI: 1.04–2.34, *p* = .032). However, no such associations were observed between the DBil and mortality risk. Moreover, the sensitivity analysis showed the same results after excluding patients with less than 1-year follow-up period (data shown in the supplemental file for Table 3).

**Table 2.** Multivariable adjustments for the survival analysis of PD patients.

	Number	Crude HR (95% CI)	Multivariate HR (95% CI)		
			Model 1	Model 2	Model 3
TBil					
≤3.3 µmol/L	349	1.00 (Ref)	1.00 (Ref)	1.00 (Ref)	1.00 (Ref)
>3.3 µmol/L	391	1.46(1.07–2.00)	1.40(1.02–1.92)	1.42(1.03–1.94)	1.69(1.11–2.56)
*p* Value		.018	.039	.031	.014
IBil					
≤1.5 µmol/L	295	1.00 (Ref)	1.00 (Ref)	1.00 (Ref)	1.00 (Ref)
>1.5 µmol/L	445	1.442(1.044–1.991)	1.444(1.045–1.995)	1.49(1.08–2.06)	1.56(1.04–2.34)
*p* Value		.026	.026	.017	.032
DBil					
≤1.4 µmol/L	294	1.00 (Ref)	1.00 (Ref)	1.00 (Ref)	1.00 (Ref)
>1.4 µmol/L	446	1.36(0.97–1.89)	1.24(0.89–1.74)	1.18(0.84–1.66)	1.50(0.94–2.39)
*p* Value		.073	.21	.338	.09

Model 1: adjusted for age and sex.

Model 2: adjusted for age, sex, diabetes, cardiovascular disease, and hypertension.

Model 3: adjusted for age, sex, diabetes, cardiovascular disease, hypertension, estimated glomerular filtration rate (eGFR), albumin level, and hemoglobin.

PD: peritoneal dialysis; HR: hazard ratio; Ref: reference group; TBil: total bilirubin; IBil: indirect bilirubin; DBil: direct bilirubin.

**Table 3.** The sensitivity analysis of multivariable adjustments for the survival analysis of PD patients.

	Crude HR (95% CI)	Multivariate HR (95% CI)		
		Model 1	Model 2	Model 3
TBil				
≤3.3 µmol/L	1.00 (Ref)	1.00 (Ref)	1.00 (Ref)	1.00 (Ref)
>3.3 µmol/L	1.58 (1.10–2.28)	1.54 (1.07–2.23)	1.55 (1.07–2.24)	1.76 (1.11–2.77)
*p* Value	.014	.022	.020	.016
IBil				
≤1.5 µmol/L	1.00 (Ref)	1.00 (Ref)	1.00 (Ref)	1.00 (Ref)
>1.5 µmol/L	1.57 (1.08–2.29)	1.59 (1.09–2.32)	1.65 (1.13–2.41)	1.78 (1.14–2.80)
*p* Value	.019	.016	.01	.012
DBil				
≤1.9 µmol/L	1.00 (Ref)	1.00 (Ref)	1.00 (Ref)	1.00 (Ref)
>1.9 µmol/L	1.32 (0.93–1.89)	1.20 (0.83–1.73)	1.21 (0.84–1.74)	1.35 (0.88–2.06)
*p* Value	.126	.329	.313	.164

Model 1: adjusted for age and sex.

Model 2: adjusted for age, sex, diabetes, cardiovascular disease, and hypertension.

Model 3: adjusted for age, sex, diabetes, cardiovascular disease, hypertension, estimated glomerular filtration rate (eGFR), albumin level, and hemoglobin.

PD: peritoneal dialysis; HR: hazard ratio; Ref: reference group; TBil: total bilirubin; IBil: indirect bilirubin; DBil: direct bilirubin.

The patients were divided into two groups according to the optimal cutoff value which obtained from receiver operating characteristic analysis.

## 4. Discussion

In this retrospective cohort study, we investigated the relationship of the levels of the three types of serum bilirubin (TBil, DBil and IBil) with the 5-year all-cause mortality of PD patients. We found that the TBil and IBil levels were significantly associated with 5-year all-cause mortality among PD patients. In addition, we observed a significantly higher mortality rate in PD patients with higher levels of serum TBil or IBil than in patients with lower levels of serum TBil and IBil. However, no such associations were observed between the DBil and mortality risk. These results were confirmed in sensitivity analyses after excluding patients with follow-up of less than 1 year. Besides, we also found that chronic glomerulonephritis was the main cause leading to ESRD and CVD was the main cause of death among PD patients, which were consistent with other previous studies [19,20].

Studies have indicated that bilirubin is not merely an end product of heme degradation but is also an effective antioxidant that effectively scavenges peroxyl radicals and suppresses the oxidation of lipids and lipoproteins [5,7,21,22]; in addition, a previous study showed that OS is one of the causal mechanisms of renal dysfunction and increases as renal dysfunction progresses as a result of increased oxidant activity and reduced antioxidant capacity [23], which indicates that bilirubin may protect renal function. In addition to being an antioxidant, bilirubin also has anti-complement properties that protect against inflammation [24]. Furthermore, bilirubin can inhibit endothelial adhesion molecules, such as E-selectin, vascular cell adhesion molecule 1 and intercellular adhesion molecule 1 [25], and an animal experiment indicated that bilirubin suppresses atherosclerotic plaque formation by disrupting endothelial vascular cell adhesion molecule 1- and intercellular adhesion molecule-1-mediated leukocyte migration through the scavenging of reactive oxygen species signaling intermediaries, which accounts for the apparent cardioprotective effects of bilirubin [26].

In addition, Chen et al. [27] and Temme et al. [28] found that compared with the lower bilirubin group, the higher bilirubin group had a lower all-cause mortality risk. However, neither of these studies provided detailed information about the serum bilirubin, such as whether the values represented the DBil or IBil. In contrast, our study showed that among PD patients, the higher TBil group had a higher mortality risk than that of the lower TBil group, which is consistent with a study by Yang et al. [16], which indicated that PD patients with a high TBil ( > 0.6 mg/dl) had higher all-cause mortality. However, this study did not focus on the associations between the DBil or IBil and the mortality risk of PD patients. Moreover, a large nationwide retrospective long-term cohort study also showed that a higher TBil level was associated with higher mortality risk [14]. There are some potential reasons that can explain this difference. First, in our cohort study, the follow-up time was 30.6 ± 20.6 months, whereas the other studies had follow-up times of more than 10 years. Second, we included PD patients in our study, whereas Chen et al. [27] assessed the association of bilirubin with mortality in HD patients. Third, all of our study population was Asian, while the population enrolled in the study of Temme et al. [28] was Belgian, who cannot represent Asians well. We showed above that the findings of other studies of Asian populations are consistent with ours [14,16]. These factors may have caused the variations between our results and those of others and there are other possible explanations for these different results. For example, serum bilirubin levels may be affected by lifestyle habits, such as exercising, drinking, smoking, and fasting. These contradictory results should be further elucidated in future studies.

In addition to the TBil, we also investigated the effects of the DBil and IBil levels on the all-cause mortality of PD patients. To the best of our knowledge, no studies have reported the association of IBil with mortality in PD patients until now. Only a very limited number of studies have found that high serum IBil levels were independent protective factors for the development of diabetic nephropathy and CKD in patients, but there was no significant association among these factors in the general population [11,15,29]. Interestingly, we found that a higher serum IBil level was a risk factor for mortality in PD patients. The mechanisms driving the association between high IBil levels and higher risk for PD patients need to be elucidated. A previous study showed that the TBil is the sum of the DBil and IBil and the IBil accounts for a high proportion of the serum bilirubin (approximately 96.4 ± 2.0%) [30], so we speculate that the serum IBil has large effects on PD patients. Although IBil possesses potent antioxidant, anti-inflammatory, complement inhibitory and possibly lipid-lowering properties, the IBil also has neurotoxic properties [10]. Moreover, an extremely elevated bilirubin level is indicative of a pathological process and translates to a higher mortality rate. These might contribute to the mechanisms underlying our finding. However, the exact mechanisms need to be further elucidated in future studies.

The IBil is extremely poorly water-soluble and it is strongly bound to albumin in plasma [31]; in contrast to the IBil, the DBil is soluble in plasma and is only weakly bound to albumin and is thus more readily available in an active form compared to the IBil [24]. A previous study showed that compared to the lower DBil group, moderate DBil levels are associated with an increased risk of all-cause mortality in acute coronary syndrome (ACS) patients afterthe percutaneous coronary intervention [32]. Nevertheless, we failed to find an association between the level of the DBil and the all-cause mortality risk. This may be due to the differences in the populations that prevent the results derived from ACS patients from being generalized to PD patients.

The present study had some limitations. First, this was a retrospective study, which can only reveal associations but not causality. Second, we only had information regarding all-cause mortality, but we did not know the exact cause of death of the PD patients; however, based on clinical experience, most dialysis patients die from cardiovascular events. Third, in this study, we only examined the baseline data, without considering the changes that occurred during the follow-up period. Fourth, because of the limited sample size, the potential risk factors were not all adjusted for in this cohort study. Hence, the effects of residual confounding factors could not be completely eliminated. Our future studies will address these issues.

## 5. Conclusions

Our findings suggest that the TBil and IBil levels were significantly associated with 5-year all-cause mortality among PD patients. However, no such associations were observed between the DBil and mortality risk.
